# Towards an agent based traffic regulation and recommendation system for the on-road air quality control

**DOI:** 10.1186/s40064-016-3282-2

**Published:** 2016-09-20

**Authors:** Abderrahmane Sadiq, Abdelaziz El Fazziki, Jamal Ouarzazi, Mohamed Sadgal

**Affiliations:** 1 Computer Systems Engineering Laboratory, Faculty of Sciences, Cadi Ayyad University, 4000 Marrakech, Morocco; 2 Laboratoire Physico-Chimie des Matériaux et Environnement (LPCME) URAC 20, Faculty of Sciences, Cadi Ayyad University, 4000 Marrakech, Morocco

**Keywords:** Air quality management, Multi-agent systems, Traffic regulation and recommendation, Dijkstra algorithm, Hadoop, Artificial neural networks

## Abstract

This paper presents an integrated and adaptive problem-solving approach to control the on-road air quality by modeling the road infrastructure, managing traffic based on pollution level and generating recommendations for road users. The aim is to reduce vehicle emissions in the most polluted road segments and optimizing the pollution levels. For this we propose the use of historical and real time pollution records and contextual data to calculate the air quality index on road networks and generate recommendations for reassigning traffic flow in order to improve the on-road air quality. The resulting air quality indexes are used in the system’s traffic network generation, which the cartography is represented by a weighted graph. The weights evolve according to the pollution indexes and path properties and the graph is therefore dynamic. Furthermore, the systems use the available pollution data and meteorological records in order to predict the on-road pollutant levels by using an artificial neural network based prediction model. The proposed approach combines the benefits of multi-agent systems, Big data technology, machine learning tools and the available data sources. For the shortest path searching in the road network, we use the Dijkstra algorithm over Hadoop MapReduce framework. The use Hadoop framework in the data retrieve and analysis process has significantly improved the performance of the proposed system. Also, the agent technology allowed proposing a suitable solution in terms of robustness and agility.

## Background

The majority of air pollution causes are originated from a direct impact of human activities, including the road vehicle use. Thus, transport, especially road traffic, is a major source of air pollution in most of the cases. Road traffic associated air pollution comes mainly from burning fossil fuels. The objective of this work is to improve the on-road air quality through the use of pollution data for the generation of recommendations to drivers, in order to encourage them to take alternative paths while avoiding the most polluted road segments (Namoun et al. 2013; Zahmatkesh et al. 2015).

In this paper, we propose the use of a traffic regulation and recommendation system able to establish the air quality indexes in urban areas, generates recommendations to the users’ and indicate the right paths to drivers based on the calculated index and path properties, to both bypass the polluted paths and also avoid generating high levels of pollutants on the alternative paths of a road network. The problem is distributed geographically and highly dynamic from the fact that the air quality levels change over time and the exact number of users which demand recommendations and then the number of vehicles to redirect to alternative paths is unknown. To overcome this problem, we adopt the MAS paradigm (Sokolova and Fernandez-Caballero 2009; Ćirić et al. 2013), a set of algorithms over a distributed framework and Murena method (Murena 2004) to generate the required pollution indexes. The system calculates the pollution level and provides recommendations and vehicles rerouting plan to reassign the traffic flow in a way to ameliorate the on-road air quality. For the data analysis, we use an online analytical processing (OLAP) tool (Foster et al. 2005; Muhammad 2010). We also proposed the use of Dijkstra as a shortest path algorithm (Namoun et al. 2013; Zahmatkesh et al. 2015). This algorithm has been widely used in road management systems, with variations to improve its performance (Fan and Shi 2010).

In order to predict air pollution level near roads we use the available meteorological data and the gathered pollutants datasets in the ANN based modeling process. The prediction results can be used to give the final users and the competent authorities more valuable recommendations. In this paper the case study focused on the ozone prediction.

Moreover, due to large amounts of data, the processing task has become a great challenge. To address this problem we suggest the use of the Hadoop framework to ensure a great flexibility and speed and make needed algorithms applicable to large scale data.

The proposed approach is illustrated over a few sections starting with a brief literature review followed by an overview of the proposed system and the road network modeling details in "Related works" and "The traffic regulation for air quality optimizing" sections. The data conceptual modeling is described in "Data conceptual modeling and implementing" section. In "ANN for air pollutant levels prediction" and "Data analysis process" sections are devoted to the air pollutant levels prediction by using ANN and the MapReduce based data analysis process. Section "Path finding in the transport network" presents the weighted road network generation and the path finding process. In "The multi-agent architecture" section is dedicated to the multi-agent system description. In Case study and results" section a case study and the experimental results are presented, followed by a conclusion and perspectives in "Conclusion" section.

## Related works

Multi-agent based systems have been considered as an efficient tool for large-scale system such as intelligent traffic and air quality management (Jin and Jie 2012). The main task of such a system is to support road managers in traffic management tasks and ameliorate the on-road air quality. Many works propose the use of the MAS technology in traffic control and management systems, such as (Namoun et al. 2013) which propose an integrated approach for modeling transport infrastructure and optimizing transport in urban areas in order to reduce carbon-dioxide emissions. The authors have used an improved version of the Dijkstra algorithm as a graph search algorithm. This algorithm has a powerful potential to solve the problems of traffic congestion, which form a road network, which is the collection of graphs. The suggested solution combines the benefits of a MAS and real time traffic information forecasting. Authors in (Ivo et al. 2011) have proposed the TraSMAPI platform that provides an integrated MAS framework which supports the communication between agents and manages the agents’ activity flow. It also provides a statistics module which is responsible for the data analysis and decision making. Sokolova and Fernandez in (Sokolova and Fernandez-Caballero 2009) have also presented an agent based decision support system for the environmental health impact assessment. The system architecture is divided into three levels: data gathering, data mining and decision making. The used data are gathered from multiple heterogeneous data sources (e.g. indexes of traffic). The analysis and design phases were made based on Prometheus methodology.

Many projects have also addressed the issue of air quality data integration; such as the Appetise project (Matejicek 2005), which the objective is to produce a pollution database fed with data gathered from different sensors distributed over the study area. These data are combined with other related data like meteorological records and used in the spatial modeling and data analyzing process.

Machine learning tools such as ANN has been frequently used to predict air quality and pollution levels using a set of inputs, like pollutant concentrations, meteorological data and the available traffic information (Fontes et al. 2013; Ignacio et al. 2011). Moreover, many research works have been dedicated to implementing of such computationally expensive algorithms and analysis tasks on parallel or distributed computing systems such as Hadoop (Apache.org 2014). The use of such Big data tools can help enhancing the existing information systems and improving their performance (Zhao et al. 2011). Das and Mohapatro (2014) have proposed a study on Hadoop’s MapReduce framework integration with data warehouses built using the traditional SQL based data warehousing system. The aim is to benefit from the performance and the fast parallel processing power of these tools.

## The traffic regulation for air quality optimizing

### Overview

The Objective behind this system is to propose a solution for traffic regulation based on air quality, meteorological and contextual collected data. A set of algorithms is used over these data filtering systems that analyses data gathered from local station’s sensors and external data sources which contains other needed data (Meteorological parameters, Geographical data and boundary conditions) and adds the intelligence of immediate contextual parameters which we can retrieve from users’ devices (e.g. Smartphone), such as time of day, location, speed, direction and weather. The resulting information will then be used to address the traffic regularization issue. As a result, users receive more relevant information and recommendations that are based on a combination of their historical preferences and contextual parameters.

Pollution data are collected from the on-road monitoring sensors and used to calculate an Atmospheric Pollution Index (API) (Murena 2004) which is used to determine the quality of air in a simple manner and provide information that can be understood by the general public. The API is based on hourly pollutant measurements comprising of Sulfur dioxide (SO_2_), Ozone (O_3_), Nitrogen dioxide (NO_2_), Atmospheric particulate matter (PM10) and Carbon monoxide (CO).

**Fig. 1:**
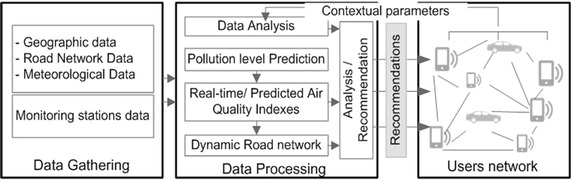
Overview of the proposed system components

Another important functionality of the system is the road graphs generation and calculation of each graph segments cost. The cost is then used to find the most environment-friendly routes for a particular user. The Cost of each segment is determined by using a number of variables such as API value and the path segment lengths. Other persistent information about segments may also be taken into account (e.g. Number of lanes). In addition, the proposed system uses the gathered historical pollution data and meteorological records to predict the pollutant levels based on the use of ANN. Figure 1 illustrates an overview of the system structuring.

### The traffic regulation process

**Fig. 2:**
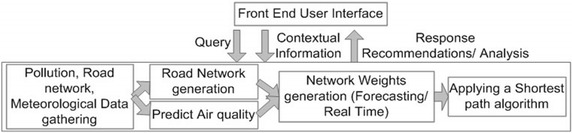
The traffic regulation and recommendation generation process

The traffic regulation and recommendation generation process is divided into the following steps (see Fig. 2):

- The pollution and traffic data gathering.
- The users contextual information integration.
- The road network generation.
- Data processing and the network weights calculation.
- Applying the chosen shortest path algorithm.

### Road network modeling

**Fig. 3:**
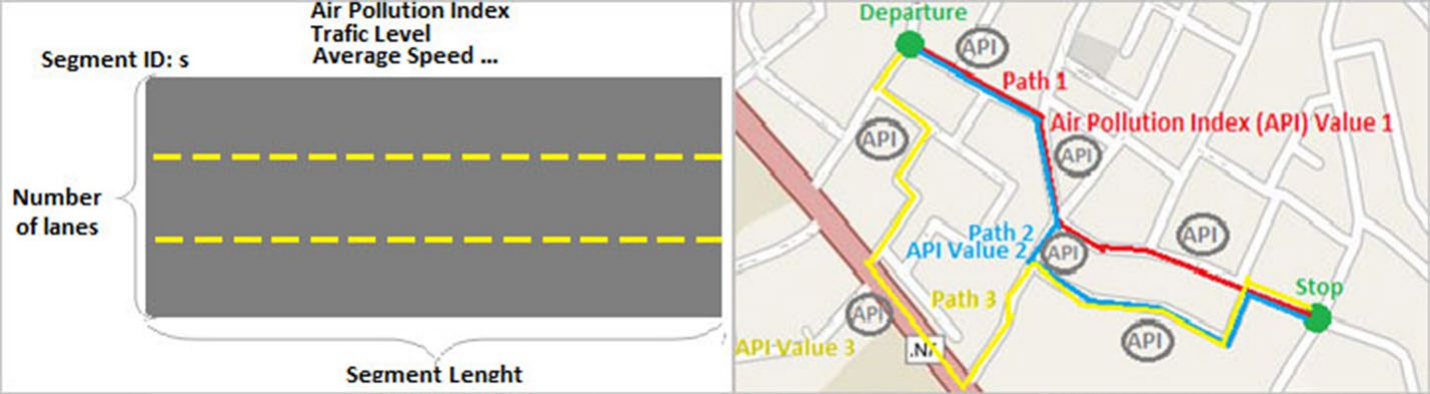
Road segment details (*left*) and an example of a road network (*right*)

The traffic flow is the result of interaction between the transport demand and the transport supply. The road network of the studied area represents the transport supply. It’s described by the road segments and its intersections. In this system we simulate the road segments for which physical properties are collected from urban and traffic management centers and delivered to the system. The gathered information, such as segment data is used in the weighted graph generation (Fig. 3).

**Fig. 4:**
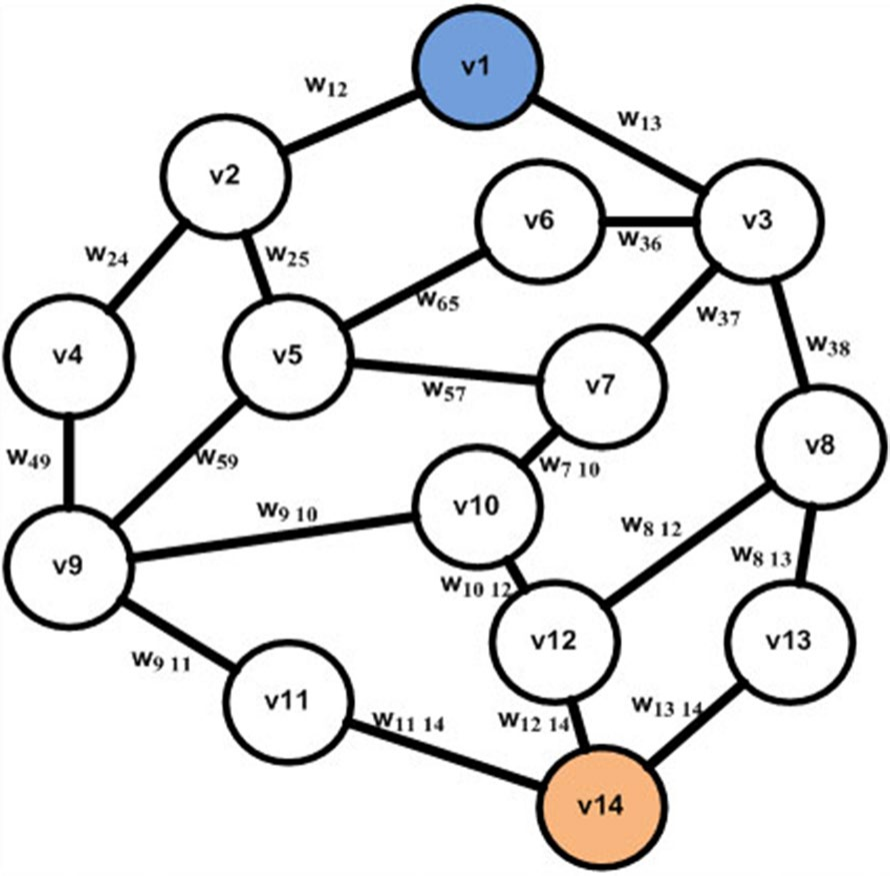
Example of a two directed edge based road graph

The network is formalized by a planar graph, in which the roads are represented by a set of arcs and the junction points by nodes. Arcs can be characterized by features such as: Pollution level, length, traffic capacity and number of lanes. Nodes are associated with characteristics specific to intersections, such as: traffic direction, the type signaling, etc. The cartography is represented by a weighted graph G = (V, E) where V is a set of vertices representing and E = V × V is a set of edges e = (v_i_, v_j_). Each segment joining adjacent vertices is represented by either one or two directed edges. The edge weight w_ij_ between two vertices v_i_ and v_j_ is a dynamic factor, which is used in order to represent properties related to the edge (v_i_, v_j_). Figure 4 shows an example of road graph.

## Data conceptual modeling and implementing

Over time, the available analysis data volumes reach critical sizes. For this, we proposed the use of Hadoop as a Big Data management tool. This solution has many advantages such as high flexibility and scalability. It also offers a cost effective storage solution and allow considering new approaches for data warehousing, especially from the multidimensional data management point of view (Cuzzocrea et al. 2013).

In the other hand, a big amount of needed data is stored mainly in relational databases which are based on the star schema (traditional data warehouses) (Dehdouh et al. 2014). Hence our interest in this section in transforming the required relational conceptual model into a “Not only SQL” (NoSQL) schema and exactly into a column-oriented schema (Chevalier et al. 2015); given that Hadoop HBase which is used in the development process is a distributed column-oriented database. For this, we define a set of automatic mapping rules, translating from the relational conceptual level to the Hbase logical model.

In this work, all data have to be extracted and stored into the HBase. It is a database with high reliability, column storage, high performance and scalability and based on the Hadoop distributed file system (HDFS). Its goal is the hosting of very large tables with billions of rows and millions of columns atop clusters of commodity hardware (Apache.org 2014).

**Fig. 5:**
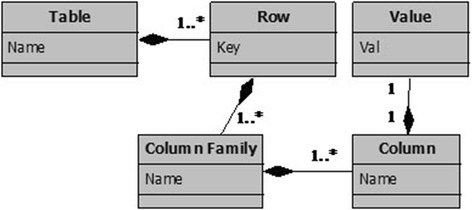
Representation of column-oriented database concepts

As most of column-oriented databases, HBase is structured into a set of tables composed of a set of rows and whose physical storage is organized by groups of columns called column families; a column family can contain a very large number of columns. For each row, a column exists if it contains a corresponding value. Through the HBase feature of column-oriented store and versioning, the time-series data sets are built based on the primary key Row-key and timestamp. Figure 5 depicts a Unified Modeling Language (UML) class diagram representing the column-oriented database components.

**Fig. 6:**
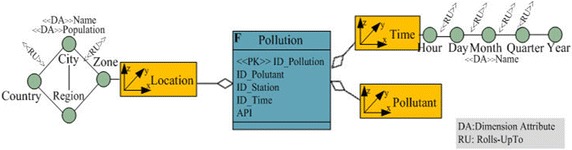
Example of the multidimensional conceptual schema

In this paper, the required pollution data conceptual model is presented as a star schema. This schema is composed of three dimensions (Time, Location and Pollutant) and a fact that represents the API records (Measure). Figure 6 illustrates the conceptual model.

The aim is defining a mapping between the relational star schema and the column-oriented model. For this, each element of the conceptual model (Facts, dimensions, attributes, etc.), is transformed into the corresponding concept in the target model according to the following set of transformation rules:

- Each Fact and its associated dimensions (Conceptual star schema), is transformed into a table.
- Each Fact is transformed into a Column Family into the corresponding table (Table with the Fact name) and the fact measures are the column of this column family.
- Each Dimension of the conceptual model is transformed into a column family in which the columns are the dimension attributes.

**Fig. 7:**
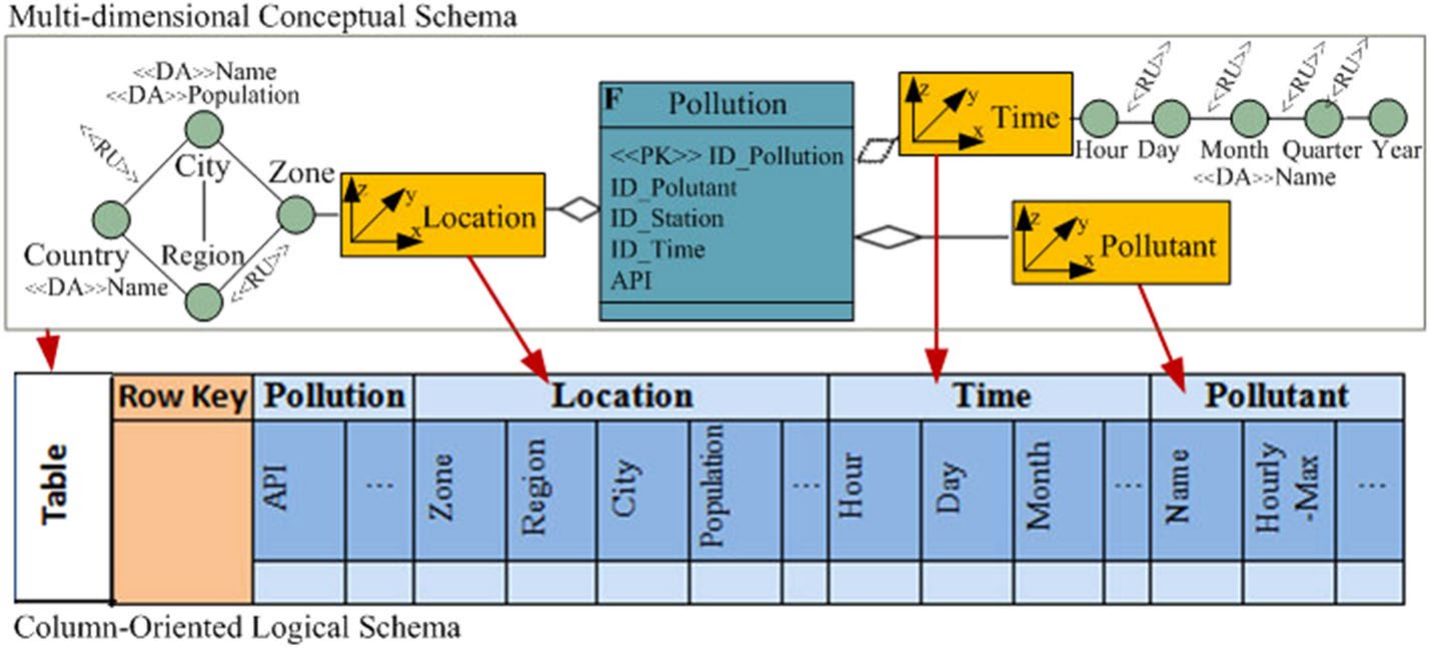
The mapping between Multidimensional conceptual model and column-oriented model

Figure 7 illustrates the relation between the two model concepts.

## ANN for air pollutant levels prediction

The on road air pollution forecasting is performed by using ANN’s. ANN are mathematical models inspired by the functioning of nervous systems, which are composed of a set of interconnected artificial neurons. These neurons can be associated in many different ways, depending on the characteristics of the issue to address. The air pollution prediction system is considered as a system that receives information from distinct set of inputs and produces a specific output (Russo et al. 2013).

The proposed network has been used for predicting pollutants concentration using a 2 years data which contains hourly concentration of different pollutants and meteorological records. This prediction process is based on three stages. The data extracting stage, in which the objective is to define the most significant data for the learning phase. The second phase is learning stage. It aims to find the optimal configuration of hidden layers, the transfer function, and the performance index. The objective during this stage is to minimize the prediction error. The third stage is the prediction part in which we predict the pollutant concentration for a given time and location from a previously calibrated neural network during the learning phase.

The Multilayer Perceptron (MLP) is an example of an artificial neural network that is used extensively for the solution of a number of different problems, including pattern recognition and interpolation. It can be seen as a function transforming the input space *X* into the output by processing every input signal by convenient weights into neurons located in the hidden layer, which transform the input by using specific activation functions, considering also the bias (Zhang 2000). They recombine frequently in a linear way those hidden outputs by convenient weights and bias as shown below:

1 $$f(x) = G\left( {b^{(2)} + W^{(2)} (s(b^{(1)} + W^{(1)} x))} \right)$$

With bias vectors $b^{(1)}$ and $b^{(2)}$; weight matrices $W^{(1)}$ and $W^{(2)}$ and activation functions $G$ and $s$.

Artificial neural network (MLP in our choice) models can treat multivariable problems. They have the aptitude to describe non-linear relationships such as that supervising ozone production. In this paper, we use ANN for modeling the ozone level by applying the artificial neural networks modeling. This method is widely available to extract those non-linear features of the relationship that a regression model might overlook. The ozone formation is a well-known phenomenon resulting from complex chemical reactions of nitrogen oxides and organic species in the presence of solar radiation (Ozbay et al. 2011). Both precursor emissions and meteorological conditions have important roles in this formation mechanism. Thus, in the ozone modeling process we use a set of meteorological data in addition to pollutant data.

## Data analysis process

**Fig. 8:**
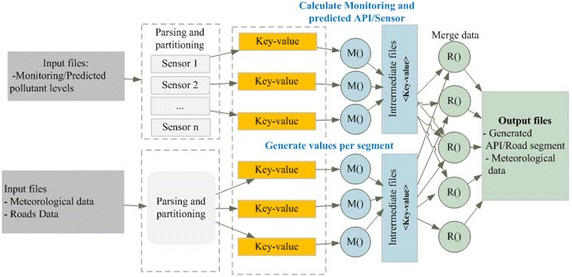
The second stage of the MapReduce process

The weighted graph generation and traffic regulation processes use a multi-phase MapReduce process to get emissions of various time resolutions on the different addressed road segments (Zhao et al. 2011). In the first MapReduce phase, the predicted and gathered monitoring data are loaded from the Hadoop HBase and used to calculate the pollution sub-indexes by applying the Murena method (Murena 2004). Geographical and Meteorological data are loaded in order to generate the final API in each road segment and recommendations for users. Meteorological data are also used in order to provide more information for road users. First, we perform a data cleaning process using a single MapReduce phase. In the second Map stage (see Fig. 8), we use the cleaned data set files to calculate the pollutant sub-indexes by applying the Murena method (Murena 2004). At the same time we use the meteorological and the road network data to generate the final API for each road segment (Zhao et al. 2011; Fang et al. 2014). The intermediate results are stored into the output databases.

**Fig. 9:**
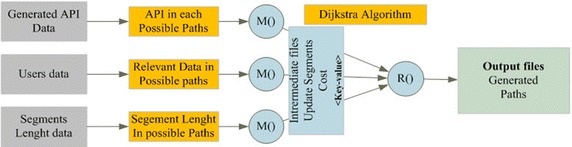
The third stage of the Map and Reduce process

In the third Map phase (Fig. 9) the API vales are combined with users’ data and road segment’s data in order to generate the weighted network. The Map stage uses the pair of segment identifier, and timestamp as the key and the API as well as edge length as value. During this MapReduce stage the Dijkstra algorithm is applied in order to generate three shortest paths in response to each user query. In the Reduce phase, the paths with the same key are cumulated together.

## Path finding in the transport network

### Weighted network generation

The weights of the network segments are calculated by combining the API records and the segment lengths. Murena method (Murena 2004) was adopted to calculate the API. The evaluation of the API at stations for a pollutant *p* (*PI*_*s*,*p*_) is carried out by a linear interpolation between the reference scale values reported in Table 1 and is given by:

2 $$PI_{s,p} = \left[ {\frac{{PI_{hi} - PI_{lo} }}{{BP_{hi} - BP_{lo} }}\left( {C_{p} - BP_{lo} } \right) + PI_{lo} } \right]_{s,p}$$

where $PI_{s,p}$, the value of the pollution index for a pollutant *p* at the site *s*; $BP_{hi}$, the lowest break-point of a pollutant *p* that is greater than or equal to *C*_*p*_; $BP_{lo}$, the highest break-point of a pollutant *p* that is lower than or equal to; $PI_{hi}$, the *PI* value corresponding to *BP*_*hi*_; $PI_{lo}$, the *PI* value corresponding to *BP*_*lo*_; $C_{p}$, the pollutant *p* daily concentration.

**Table 1:** Breakpoints (µg/m^3^ for all pollutants and mg/m^3^ for CO) for the proposed API (Murena 2004)

Pollution level	API	PM_10_	NO_2_	CO	SO_2_	O_3_
Unhealthy	85–100	238–500	950–1900	15.5–30	500–1000	223–500
Unhealthy for sensitive groups	70–85	144–238	400–950	11.6–15.5	250–500	180–223
Moderate pollution	50–70	50–144	200–400	10–11.6	125–250	120–180
Low pollution	25–50	20–50	40–200	4–10	20–125	65–120
Good quality	0–25	0–20	0–40	0–4	0–20	0–65

An individual score is assigned to the level of each pollutant and the final API index is equal to the highest sub-index determined for each of the considered pollutant. The other required road segments properties are prepared and stored in internal databases. The weights of the network are updated when new forecasts are available. In this case, the system recalculates the API and dynamically updates the network with the new values. Otherwise the current weights are used to generate paths and recommendations.

### Shortest path algorithm: Dijkstra

Path finding in weighted traffic networks is the process of searching for the shortest possible road between two points. The path is represented via a set of segments to browse from a start node to the destination node. The aim is to create a road network, where the weight of each segment is a combination of the different nature of the cost of using this particular segment. As in this work we are interested in the air quality optimizing by traffic regulation, we use the on-road calculated API values and the segment lengths (Namoun et al. 2013).

Using this weighted network the system can process user queries and generates recommendations and calculate potential routes according to the Dijkstra shortest path algorithm. For a particular source node, the algorithm finds the path with the lowest cost between that node and any other node. The used function for calculating the shortest path will depend on the road segment length and the calculated API for each path segment. Thus, the value of the path can be provided using the formula:

3 $$P_{i} = \sum\limits_{i = 0}^{n} {X_{i} } \times W_{i}$$

4 $$W_{i} = \left( {C_{1} \times A_{i} + C_{2} \times L_{i} } \right)/\left( {C_{1} + C_{2} } \right)$$

where, *X*_*i*_ is a configurable weight and *W*_*i*_ is the weight of the edge number *i* and n is the potential number of edges to browse. *A*_*i*_ and *L*_*i*_ are successively the current API and length of the edge *i*. *C*_1_ and *C*_2_ are two coefficients related to each cost parameter. They can be controlled in order to optimize the weight calculation function. In this work we modify the Dijkstra’s shortest path algorithm to obtain three least polluted paths (shortest paths) in order to allow users to make their own decisions based on the provided information and their own risk evaluation. The Dijkstra algorithm is implemented based on the use of the parallel breadth first search algorithm.

## The multi-agent architecture

**Fig. 10:**
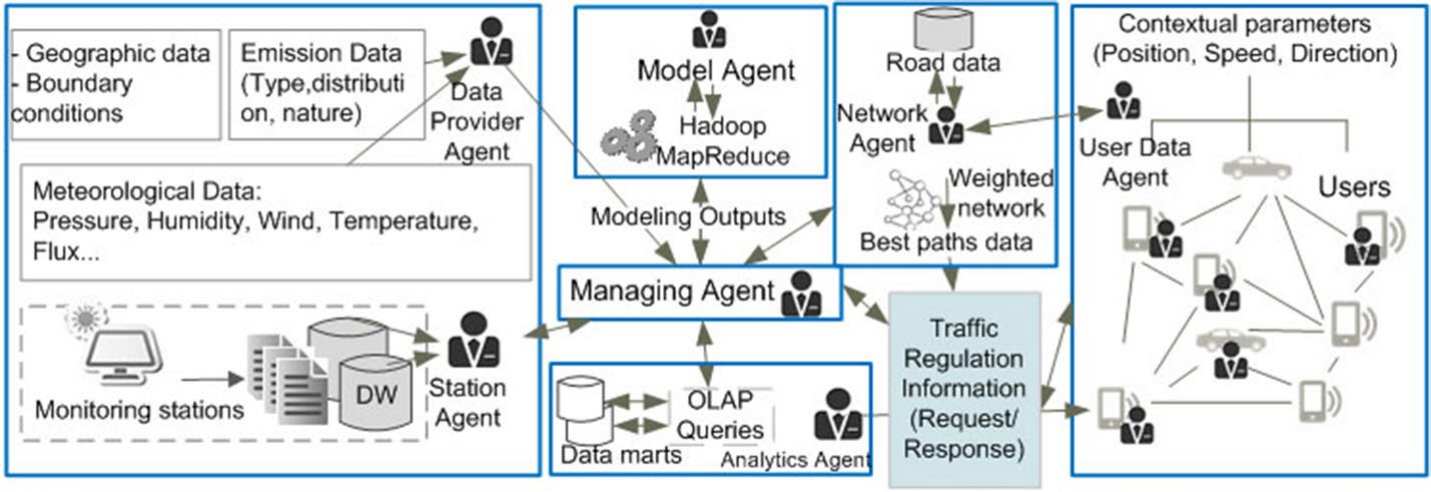
The multi-agent air quality system for traffic regulation

In this work we represent the proposed system by using a multi-agent based architecture. Thus, the system is composed of a group of autonomous agents which have the ability to set their own activities and goals and communicate via an interaction protocol in order to achieve the system main objective (Lavbic and Rupnik 2009). The main objective behind the use of MAS is to propose an adequate solution in terms of adaptability, flexibility and agility. Figure 10 shows the multi-agent system structuring.

In the development process we chose to use the Prometheus methodology to design and implement the multi-agent system. Our choice is motivated by the efficiency of message communication and lightweight nature of the framework. Prometheus methodology (Lin et al. 2007) has been developed to support the complete software development lifecycle from problem description to implementation. It offers an environment for analyzing, designing, and developing heterogeneous multi-agent systems. This methodology consists of three phases: System Specification, architectural design and the detailed design. The Prometheus Development tool is extended with the ability to generate skeleton code in the Java Agent Compiler and Kernel (JACK) agent-oriented programming language (AOS 2008) using the Prometheus Design Tool (PDT) code generator extension which maintains also synchronization between the generated code and the design when either of them changes.

The proposed system is composed of seven main agents: Station agents, Data provider agent, Model agent, Network agent, Managing agent, Users Data agent, and Analytics agent.

### Station agents

The station agents are responsible for the continuous air quality measurements gathering from the existing monitoring sensors in the study area. They are used also for data loading (to the managing agent) and data transformation and conversion of the heterogeneous retrieved data (e.g. XML files). The data provided by these different agents will allow a better understanding of the spatiotemporal evolution of air pollution.

### Data provider agent

This agent is in charge of extracting data from external data sources and provides all required inputs for the managing and model agents. It sets up data sets of needed geographical data, meteorological data, emission data, etc. (Menut et al. 2013).

### Model agent

The objective of the model agent is the quantification of the evolution of pollutants according to time on road segments, taking into accounts all available data and parameters. This agent calculates and provides an average concentration over a surface (road network) based on the use of a set of equations. It uses a single Hadoop MapReduce phase (Fang et al. 2014) to calculate the API values using the pollutant concentrations. The API values are then used for the weighted road network generation. It’s also responsible for the prediction of the air pollutant levels by using ANN. The modeling results are stored in an HBase.

### Network agent

This agent is responsible of the road paths network generation and path finding in this weighted network by searching for the shortest possible route between two road nodes. The path is represented by a set of segments to browse from a source node to the destination node.

### Managing agent

Responsible for the reliability of the whole system, and manages the operation of the individual agents, especially the station agents, model, data provider and network generation.

### User’s data agent

This agent uses the user’s devices in order gather data concerning user’s location, traffic and road conditions using sensors readings (e.g. Accelerometer, GPS).

### Analytics agent

The aim of the analytics agent is to enhance the value of both gathered and resulting data (pollutants concentrations, meteorological data, API values, graph details, etc.) by converting them into a more valuable and intelligible information for final users. It applies a fast and effective analysis and creates recommendations for users. This agent integrates the OLAP tools features (for a fast analysis of multidimensional data) and data mining methods which are more suitable for large data sets processing (Muhammad 2010). The analytics agent performs needed analysis and reports the results back to the concerned entity (e.g. managing agent).

### Agents’ interactions

**Fig. 11:**
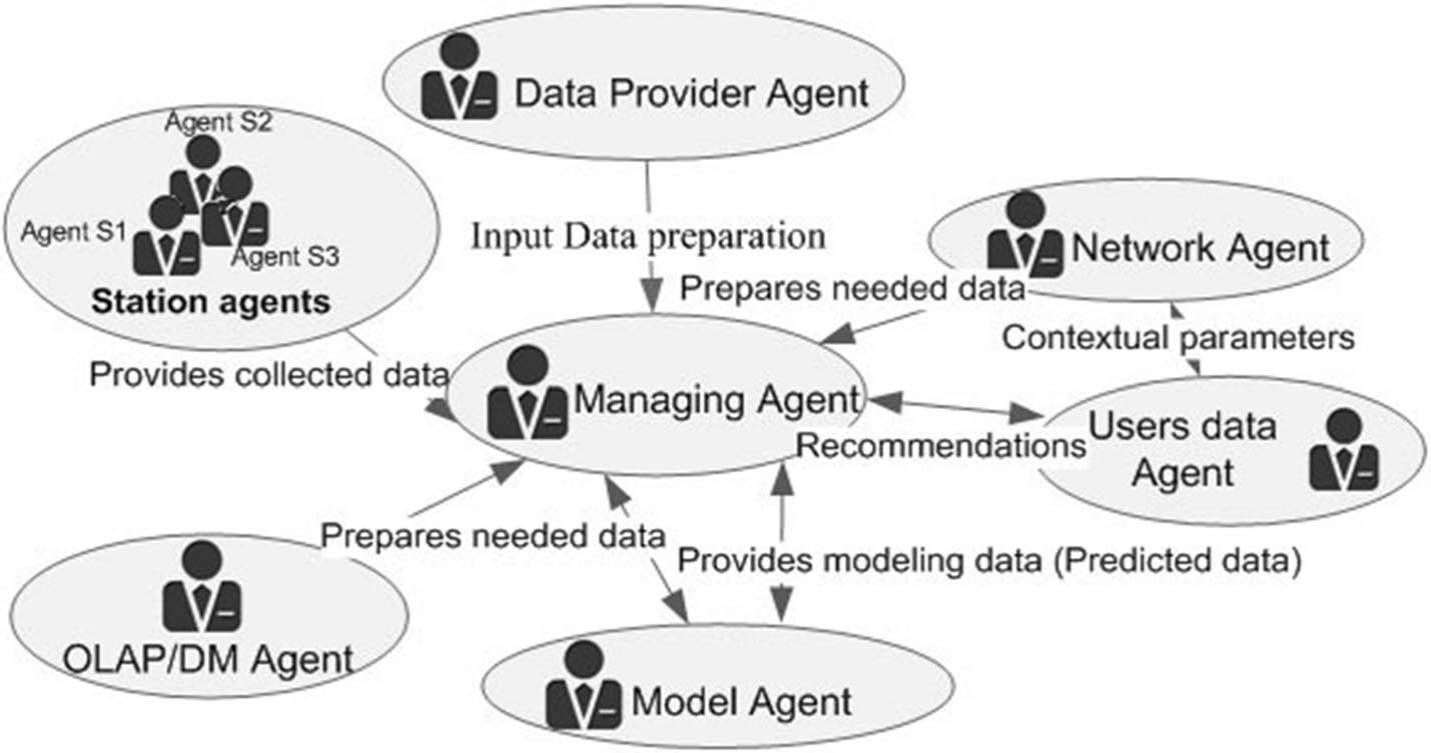
General architecture of the agent’s framework

The Managing agent ensures most communicative exchanges between the other system’s agents. The agents communicate based on a direct interaction mode, using structured messages. Such a service is provided by the FIPA-JACK extension which supports the FIPA Agent Communications Language (AOS 2008; Fernandez-Caballero and Gascuena 2010). Figure 11, illustrates an overview of the different agents and their interactions.

## Case study and results

### The study area

**Fig. 12:**
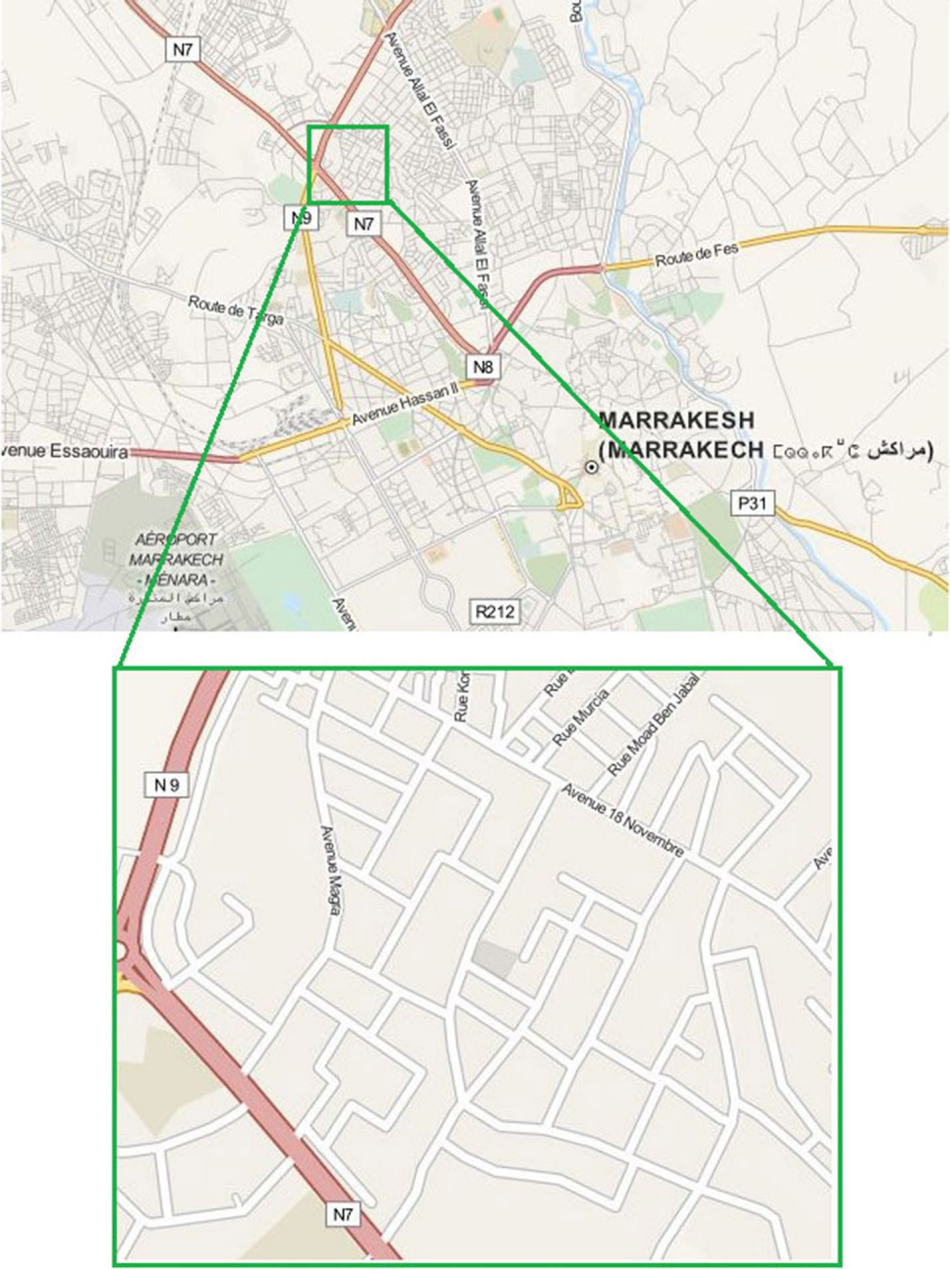
Inset map of an area of Marrakech—Morocco used for simulations (by Google Maps)

The study area used for the testing scenario in this work is located in Marrakech-City. This study area suffers from the effects of pollutants produced by vehicle exhaust systems. This study is based on a set of sensors that provide information and measures of the air pollutant concentration. It focused on air quality indexes related to the following pollutants: Sulfur Dioxide (SO_2_), Nitrogen dioxides (NO_2_), Carbon Monoxide (CO), Particulate Matter, and Ozone (O_3_). The map used for the testing scenario in this paper was cut in order to reduce the simulation time and the area considered in the simulations is represented in Fig. 12.

### Experimental data

In our experiments, we used datasets issued from different near-road air quality monitoring stations (during 2009 and 2010). The data records have hourly frequency. The available records for each monitoring station are variable due to the sensors deficient time. Each record contains 11 attribute: The five pollutants concentration (SO_2_, O_3_, NO_2_, PM10 and CO), solar radiation, Wind speed, Temperature (Celsius), Humidity ratio, Date and Time.

### API values generation

**Fig. 13:**
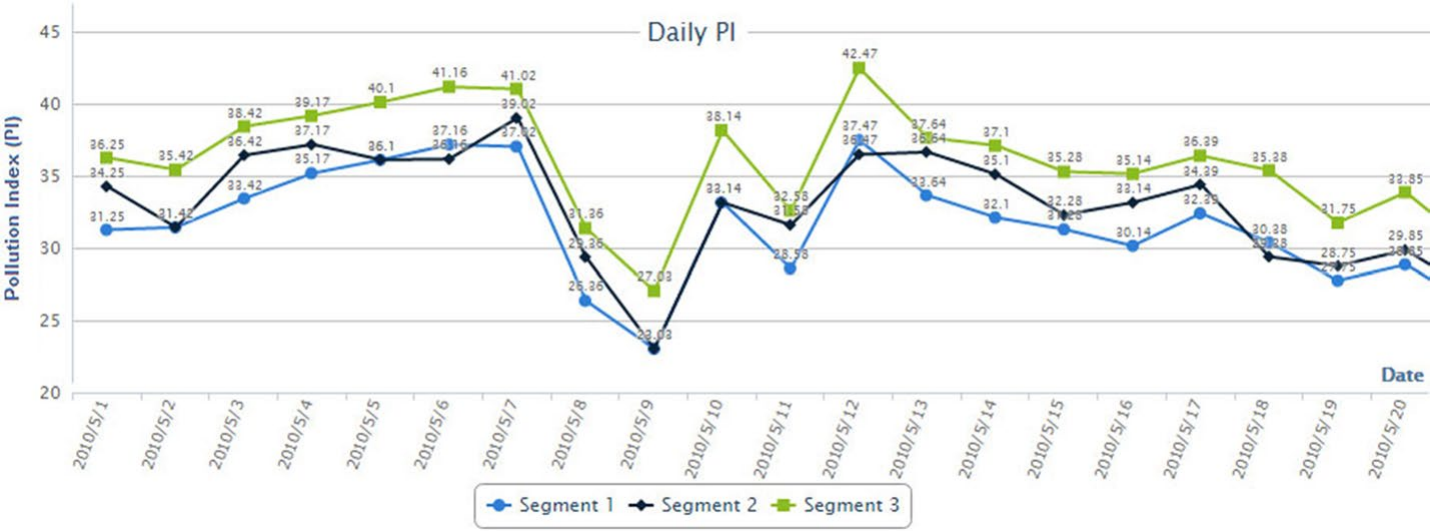
The pollution index in three segments in the simulation area

The system generates the analysis queries based on selected options from an easy to use user interface to get the resulting API. Figure 13 shows an example of a query result which allows selecting the API during May 2010 in a selected location from the study area.

### Least polluted path finding

**Fig. 14:**
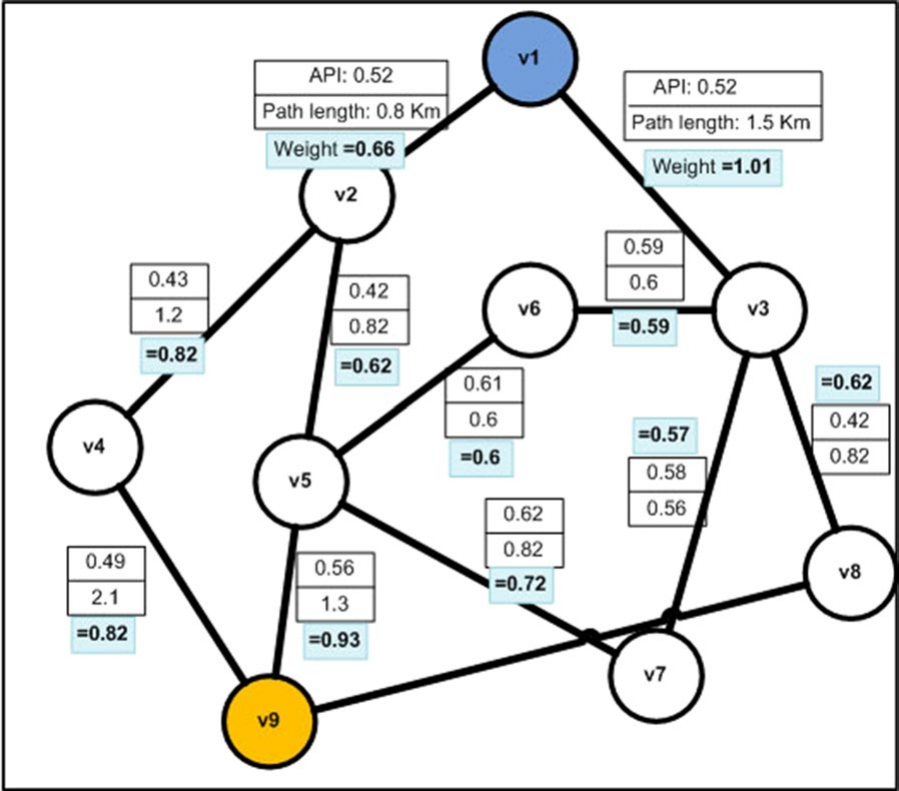
Part of the study area weighted road graph

**Fig. 15:**
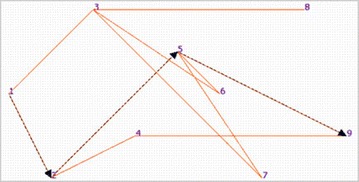
The resulting shortest path (Screenshot)

In the implemented system prototype, the road graph is generated in a specific location in the study area. For each segment in this location the system calculates or updates the weight (w) which is based on the API and paths length. The Dijkstra algorithm is then applied in order to obtain the less polluted paths. The system generates then the suitable recommendations for the road network users and competent authorities. Figure 14 describes a road network example, in which a user wants to navigate from v1 to v9. An example of the resulting shortest path is presented in Fig. 15 (Screenshot of the output).

### The ozone level prediction

**Fig. 16:**
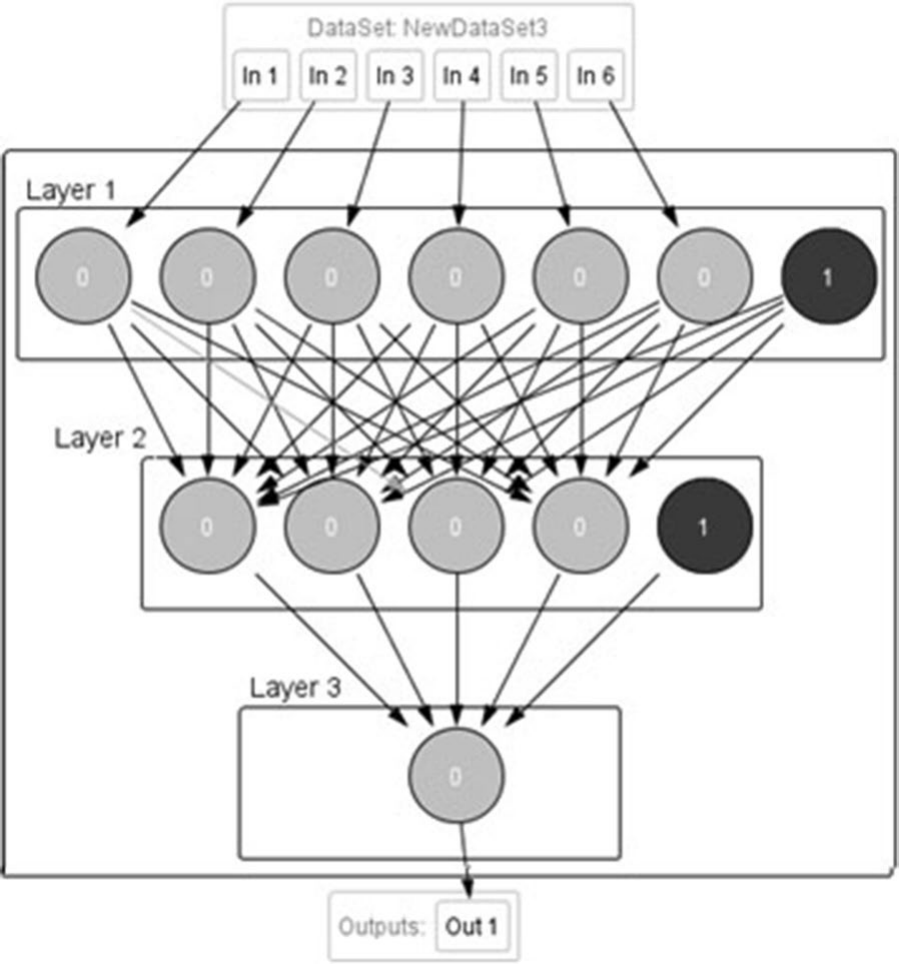
The three-layer ANN configuration (for Dawdiat station)

In this study, we used a three-layer perceptron ANN model (see Fig. 16) to predict the Ozone concentrations which are influenced by the meteorological conditions, especially temperature and solar radiation; Furthermore, the Ozone formation depends also on the NO_2_, PM10 and CO levels in the atmosphere (Ozbay et al. 2011). Thus The input parameters for ozone prediction (first layer) by MLP method are NO_2_, CO, PM10, the relative humidity (RH), the wind speed (WS), the temperature (T) (Temperature records are not available for dawdiat station) and the solar radiation (SR). The number of hidden layers and neurons in each hidden layer (second layer) are the parameters to choose in the model construction.

The last layer is the output, which consists of the target of the prediction model (O_3_ in this case study). A two-year dataset was divided into two parts: 80 % used for training the networks and the remaining 20 % employed in testing the networks.

In our case, we tested different configuration and we found that the best architecture was a fully connected configuration, linear output layer and logistic as activation functions with one hidden layer and the following hidden neurons (Table 2):

**Table 2:** Number of hidden neurons for the MLP architecture

Station	Dataset
Dawdiat (6-X-1)	4
Mhamid (7-X-1)	19
JEF (7-X-1)	13

The following Fig. 16 illustrates the ANN configuration (for Dawdiat station):

After selecting the most suitable configuration from the tenfold cross validation process it will be possible to determine the model fitness (by using training data) by calculating the adjusted coefficient of determination R^2^. The MLP model fitness is presented in Table 3.

**Table 3:** The Coefficient of determination R^2^ for the ANN

Station	Dataset
Dawdiat	0.526
Mhamid	0.532
JEF	0.672

**Fig. 17:**
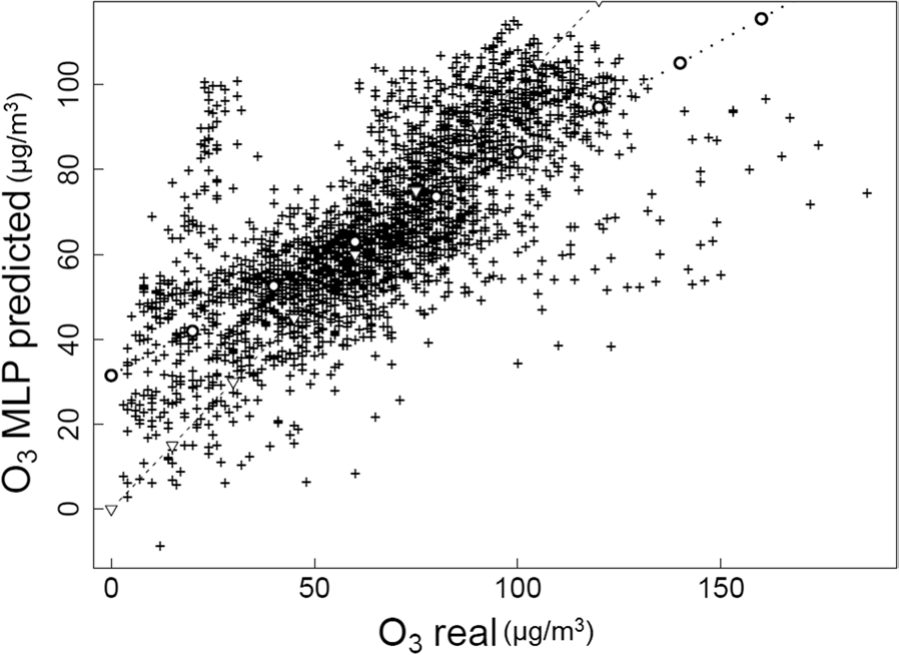
Predicted O_3_ by the MLP model against its measurements (Dawdiat station)

Figure 17, shows the scatter plot of the predicted ozone concentrations versus the corresponding observed values in dawdiat station.

## Conclusion

We have, through this paper presented the implementation of an air quality system for recommendation and traffic regulation by using distributed data gathered from different air quality monitoring stations and other contextual data. The data gathering and analysis process is based on the use of Big data tools to ensure a fast data loading, fast query processing and an efficient storage. In this work we also proposed the use of a multi-agent framework to represent the system components. The case study addresses the generation of the air pollution indexes on a specific road network and the prediction of the ozone level. These pollution indexes are then used in order to calculate the best route users can borrow. Our experimental results show that the data processing operations and the Dijkstra algorithm deployed in the large-scale data processing system are feasible and efficient. The perspectives of this work concern the use of multi-criteria decision support tools, the integration of traffic data in order to evaluate the system effectiveness regarding the reduction of the pollution level and the evaluation of the system performance when dealing with a larger amount of data.

## Abbreviations

ANN: artificial neural network
API: atmospheric pollution index
BI: break-point
CO: carbon monoxide
FIPA: foundation for intelligent physical agents
GPS: global positioning system
HDFS: hadoop distributed file system
JACK: Java Agent Compiler and Kernel
MAS: multi-agent systems
MLP: multilayer perceptron
NO_2_: nitrogen dioxide
NoSQL: not only SQL
O_3_: ozone
OLAP: online analytical processing
PDT: prometheus design tool
PI: pollution index
PM10: atmospheric particulate matter
RH: relative humidity
SO_2_: sulfur dioxide
SR: solar radiation
T: temperature
UML: unified modeling language
WS: wind speed
XML: extensible mark-up language

## References

[CR1] AOS (2008) JACK intelligent agents. Agent manual. In: Agent Oriented Softw. Pty. Ltd. www.aosgrp.com/documentation/jack/Agent_Manual.pdf. Accessed 24 Feb 2016

[CR2] Apache.org (2014) Apache Hadoop. https://hadoop.apache.org/. Accessed 02 Jan 2016

[CR3] Chevalier M, El Malki, Mohammed Akopliku A, Olivier T, Ronan T (2015) Not only SQL implementation of multidimensional database. In: International conference on data warehousing and knowledge discovery (DaWaK 2015), Springer, Valencia

[CR4] Ćirić IT, Ćojbašić ŽM, Nikolić VD (2013). Air quality estimation by computational intelligence methodologies. Therm Sci.

[CR5] Cuzzocrea A, Bellatreche L, Song I-Y (2013) Data warehousing and OLAP over big data: current challenges and future research directions. In: Proceedings of the sixteenth international workshop on data warehousing and olap, ACM, San Francisco, CA

[CR6] Das TK, Mohapatro A (2014). A study on big data integration with data warehouse. Int J Comput Trends Technol.

[CR7] Dehdouh K, Boussaid O, Bentayeb F (2014) Columnar NoSQL star schema benchmark. In: Ait Ameur Y, Bellatreche L, Papadopoulos G (eds) MEDI’2014: 4th international conference on model and data engineering, Larnaca, Cyprus, September 2014. Lecture notes in computer science (Model and Data Engineering), vol 8748. Springer, Heidelberg

[CR8] Fan D, Shi P (2010) Improvement of Dijkstra’s algorithm and its application in route planning. In: Proceedings of the seventh international conference on fuzzy systems and knowledge discovery (FSKD). Yantai, China, pp 1901–1904

[CR9] Fang W, Sheng VS, Wen X, Pan W (2014). Meteorological data analysis using MapReduce. Sci World J.

[CR10] Fernandez-Caballero A, Gascuena JM (2010) Developing multi-agent systems through integrating prometheus, INGENIAS and ICARO-T. In: Filipe J, Fred A, Sharp B (eds) ICAART 2009: international conference on agents and artificial intelligence, Porto, Portugal, January 2009. Communications in computer and information science (agents and artificial intelligence), vol 67. Springer, Heidelberg

[CR11] Fontes T, Silva LM., Pereira SR, Coelho MC (2013) Application of artificial neural networks to predict the impact of traffic emissions on human health. In: Luís C, Luís PR, José C (eds) EPIA 2013: 16th Portuguese conference on artificial intelligence, Angra do Heroísmo, Azores, Portugal, September 2013. Lecture notes in computer science (progress in artificial intelligence), vol 8154. Springer, Berlin, Heidelber, pp 21–29

[CR12] Foster D, McGregor C, El-Masri S (2005) A survey of agent-based intelligent decision support systems to support clinical management and research. In: Proceedings of the workshop on multi-agent systems for medicine, computational biology, and bioinformatics in association with the 4th international joint conference on autonomous agents and multi-agent systems, Utrecht

[CR13] Ignacio G, Jose G, Yenisse M (2011) Artificial neural network models for prediction of ozone concentrations in Guadalajara, Mexico. In: Dragana P (ed) Air quality-models and applications, InTech. http://www.intechopen.com/books/air-quality-models-andapplications/artificial-neural-network-models-for-prediction-of-ozoneconcentrations-in-guadalajara-mexico. Accessed 25 June 2016

[CR14] Ivo T, Miguel A, Rosaldo R, Eugénio O (2011) Using TraSMAPI for developing multi-agent intelligent traffic management solutions. In: Demazeau Y, Pěchoucěk M, Corchado J, Pérez J (eds) PAAMS 2011: 9th international conference on practical applications of agents and multiagent systems. Salamanca, Spain, April 2011. Advances in intelligent and soft computing (advances on practical applications of agents and multiagent systems), vol 88, Springer, Heidelberg

[CR15] Jin X, Jie L (2012). A study of multi-agent based model for urban intelligent transport systems. Int J Adv Comput Technol.

[CR16] Lavbic D, Rupnik R (2009). Multi-agent system for decision support in enterprises. J Inf Organ Sci.

[CR17] Lin P, Thangarajah J, Winikoff M (2007) AUML protocols and code generation in the Prometheus design tool. In: Proceedings of the 6th international joint conference on autonomous agents and multiagent systems (AAMAS-07), Honolulu, Hawaii

[CR18] Matejicek L (2005). Spatial modelling of air pollution in urban areas with GIS: a case study on integrated database development. Adv Geosci.

[CR19] Menut L, Bessagnet B, Khvorostyanov D (2013). CHIMERE 2013: a model for regional atmospheric composition modelling. Geosci Model Dev.

[CR20] Muhammad S (2010) Development and implementation of air quality data mart for Ontario, Canada : a case study of air quality in Ontario using OLAP tool. Lund University

[CR21] Murena F (2004). Measuring air quality over large urban areas: development and application of an air pollution index at the urban area of Naples. Atmos Environ.

[CR22] Namoun A, Marin CA, Saint Germain B et al (2013) A multi-agent system for modelling urban transport infrastructure using intelligent traffic forecasts. In: Marik V, Lastra JLM, Skobelev P (eds) Industrial applications of holonic and multi-agent systems: 6th international conference, HoloMAS 2013, Prague, Czech Republic, 2013

[CR23] Ozbay B, Keskin GA, Dogruparmak SC, Ayberk S (2011). Predicting tropospheric ozone concentrations in different temporal scales by using multilayer perceptron models. Ecol Inform.

[CR24] Russo A, Raischel F, Lind PG (2013). Air quality prediction using optimal neural networks with stochastic variables. Atmos Environ.

[CR25] Sokolova MV, Fernandez-Caballero A (2009). Modeling and implementing an agent-based environmental health impact decision support system. Expert Syst Appl.

[CR26] Zahmatkesh H, Saber M, Malekpour M (2015). A new method for urban travel rout planning based on air pollution sensor data. Curr World Environ.

[CR27] Zhang GP (2000). Neural networks for classification: a survey. IEEE Trans Syst Man Cybern Part C.

[CR28] Zhao J, Zhang J, Jia S et al (2011) A MapReduce framework for on-road mobile fossil fuel combustion CO_2_ emission estimation. In: Proceedings of 19th international conference on geoinformatics, Shanghai

